# Prevalence and comorbidities of disruptive mood dysregulation disorder

**DOI:** 10.1192/j.eurpsy.2024.948

**Published:** 2024-08-27

**Authors:** S. Riam

**Affiliations:** Arrazi University Psychiatric Hospital, Rabat Faculty of Medicine and Pharmacy Mohammed V University, Rabat, Morocco

## Abstract

**Introduction:**

Following the release of DSM-5 in 2013, a newly introduced diagnostic category emerged in psychiatric classification—the disruptive mood dysregulation disorder (DMDD). DMDD is a depressive disorder that begins in childhood and is marked by a consistently irritable or angry mood, frequently accompanied by temper outbursts that are notably severe.

**Objectives:**

It is to study the prevalence and comorbidities of DMDD in Morocco, specifically at Arrazi University Psychiatric Hospital

**Methods:**

Data were collected from youths aged 6 to 18 years who underwent a systematic assessment of symptoms. This assessment was extended to all patients consulting at Arrazi Hospital in Salé within the pediatric department over a period of four months (sample accrued from July 2023 to October 2023). The diagnosis of DMDD was established utilizing the diagnostic criteria outlined in DSM-5

**Results:**

About 31% of the young participants met the operational criteria for DMDD. Those with DMDD exhibited increased comorbidity rates with attention-deficit/hyperactivity disorder (ADHD), another Depressive disorder and conduct disorder compared to those without DMDD. Additionally, they displayed elevated symptoms of aggressive behavior, rule-breaking, social issues, anxiety/depression, attention problems, and thought problems in comparison to all other participants without DMDD. It’s noteworthy that youth with DMDD presented with at least one psychiatric comorbidity.

**Conclusions:**

Given that DMDD is accompanied by other comorbid psychiatric disorders, particularly depression and anxiety, and appears to impact familial and occupational status in adulthood, the early detection and subsequent effective treatment of DMDD symptoms are of utmost importance.

**Disclosure of Interest:**

None Declared

